# A novel de novo partial xq duplication in a girl with short stature, nonverbal learning disability and diminished ovarian reserve - effect of growth hormone treatment and fertility preservation strategies: a case report and up-to-date review

**DOI:** 10.1186/s13633-019-0071-z

**Published:** 2020-01-09

**Authors:** Francesca Parissone, Mairi Pucci, Emanuela Meneghelli, Orsetta Zuffardi, Rossana Di Paola, Stefano Zaffagnini, Massimo Franchi, Elisabetta Santangelo, Gaetano Cantalupo, Paolo Cavarzere, Franco Antoniazzi, Giorgio Piacentini, Rossella Gaudino

**Affiliations:** 1 Department of Obstetrics and Gynaecology, AOUI Verona, Verona, Italy; 2 Department of Neurological, Biomedical and Movement Sciences, Clinical Biochemistry section, University of Verona, P.le L. A Scuro, 10, 37134 Verona, Italy; 3 Department of Molecular Medicine, University of Pavia, Pavia, Italy; 4 Department of Surgical Sciences, Dentistry, Gynaecology and Paediatrics, Division of Obstetrics and Gynaecology, University of Verona, Verona, Italy; 5 Department of Surgical Sciences, Dentistry, Gynaecology and Paediatrics, Division of Child Neuropsychiatry, University of Verona, Verona, Italy; 6 Department of Surgical Sciences, Dentistry, Gynaecology and Paediatrics, Division of Paediatrics, University of Verona, Verona, Italy

**Keywords:** Chromosomal rearrangement, Xq duplication syndrome, Diminished ovarian reserve, Fertility preservation, Short stature, Recombinant growth hormone (rGH) therapy

## Abstract

**Background:**

Xq duplication is a rare condition with a very variable phenotype, which could mimic other genetic syndromes involving the long arm of chromosome X. Sometimes short stature and diminished ovarian reserve (DOR) may be present. Treatments with rGH (Recombinant growth Hormon) or with fertility preservation strategies have not been previously described.

**Case presentation:**

We present the case of a female with a novel de novo Xq partial duplication (karyotype: 46,Xder(X)(qter→q21.31::pter→qter) confirmed by array-CGH analysis. She presented with short stature, Nonverbal Learning Disability, developmental delay during childhood, severe scoliosis, spontaneous onset of menarche and irregular menstrual cycles. AMH (Anti-Müllerian Hormone) allowed detection of a preserved but severely diminished ovarian reserve with a POI (Premature Ovarian insufficiency) onset risk. She was effectively subjected to fertility preservation strategies and rGH therapy. We also reviewed other published cases with Xq duplication, reporting the main clinics characteristics and any adopted treatment.

**Conclusions:**

rGH treatment and cryopreservation in a multidisciplinary approach are good therapeutic strategies for Xq duplication syndrome with short stature and premature ovarian failure.

## Background

Duplications of the long arm of chromosome X (Xq) include intrachromosomal duplications and partial disomies/trisomies resulting from unbalanced translocations with an autosome or with a chromosome Y [1]. Complex and extended, cytogenetically visible duplications of Xq are rare and more often involve the distal Xq27-qter region [2]. To date, CGH array techniques allow even the detection of smaller imbalances. Prevalence of duplications of the distal part of chromosome Xq is yet unknown; in particular, duplications that include MECP2 gene in Xq28, responsible for neurologic impairment in these patients, are of major interest with a few hundred cases reported in literature [14, 20].

While males with dup (Xq) usually present short stature, mental retardation, feeding problems, microcephaly, facial dysmorphism, hypotonia, and hypoplastic genitalia, females with dup (Xq) may show some phenotypic abnormalities that could include short stature, developmental delay, facial dysmorphism, and gonadal dysgenesis [1, 7]. This condition could be defined as Xq Duplication Syndrome [7]. The phenotypic characteristics vary significantly in expression and severity depending on the size of duplication, the sex of the affected person, and on the genes of the chromosome duplicated segment [15]. In general, males are more likely to be severely affected by this syndrome than females bearing the same duplication, since the duplicated X chromosome is usually inactivated [1, 5].

Moreover, it is well known that the integrity and the correct functionality of the long arm of the X chromosome are required to maintain fertility. This is demonstrated by Turner Syndrome (TS), a clinical conditions with complete or partial loss of X chromosome, which is the main genetic cause of POI (Premature Ovarian Insufficiency) [21].

Our study will presents for the first time the case of a woman with a de novo duplication of Xq21.31- > qter with short stature and affected by Nonverbal Learning Disability, responsive to rGH therapy and having a preserved, even if severely compromised, ovarian activity. We also reviewed other female Xq duplication cases reported in literature in the last 35 years, highlighting the main clinical features and treatments adopted.

### Case presentation

Our patient is a 18 years old woman, the second child of a healthy and unrelated couple. Both parents were 32 years old at her birth. Her older sister was healthy. She was born small for gestational age (SGA) at 35 gestational weeks by vaginal delivery. Birth weight was 1100 g and body length was 41 cm. Developmental delay with short stature and low body weight, moderate scoliosis and a very mild intellectual disability with delayed speech have been noticed since her childhood. Spontaneous thelarche occurred at age of 11 years and menarche at the age of 14 years. The menses were always present even if irregular in rhythm. Daily subcutaneous rGH therapy at dose of 0.2 mg/kg/week was started at the age of 4 years because of SGA (clonidine GH stimulation test was: GH peak value of 11 ng/ml; arginine GH stimulation test was: GH peak value of 16 ng/mL); at that time, her weight was 11 kg (− 2.7 SD score) and her height was 90 cm (− 2.9 SD score) with a target height of 169.5 cm (+ 1.25 SD score). The 3 years follow-up growth parameters did not reveal any improvement; therefore at the age of 7 years the dose was increased to 0.3 mg/kg/week, as Variant of Turner Syndrome.

**Fig. 1:**
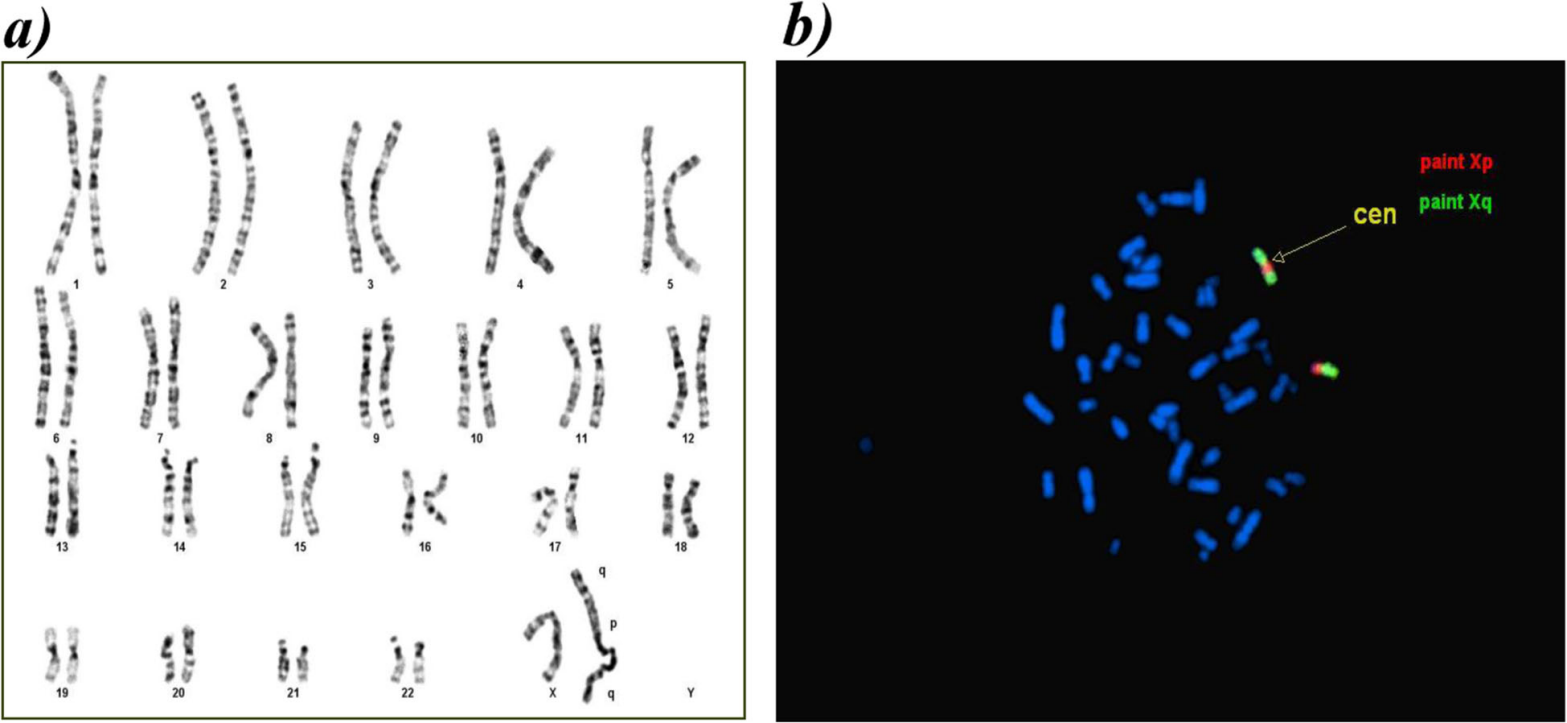
**a** Patient’s G banded karyotype shows one normal X chromosome and one aberrant X chromosome that contains a duplicated segment Xq 21.31- > qter on Xp extremity. **b** Fluorescence in situ hybridization using specific chromoprobe for Xp (green probe) and Xq arms (red probe)

Metaphase chromosome analysis from peripheral blood lymphocytes culture and GTG banding was performed using standard protocol. Cytogenetic analysis showed a female karyotype containing additional material on one X chromosome. Fluorescence in situ hybridization (FISH) with arm-specific probes (chromoprobe, Cytocell) and subtelomeric Xp and Xq probes (Vysis) allowed detection of the Xp and Xq regions on the duplicated X chromosome. The karyotype was then described as 46,X dup(X) (q21.31 → q28) (Fig. 1a and b). In order to better define the exact extent of duplication and to deepen the origin of the developmental delay and the short stature, she performed oligonucleotide-based array comparative genomic hybridization which showed partial trisomy and tetrasomy Xq [arr Xq21.31(89,594,027-89,837,847) × 3,Xq21.31(89,853,144-90,222,617) × 4,Xq21.31q28(90,254,419-155,257,082) × 3] with a 65 Mb duplication.

**Fig. 2:**
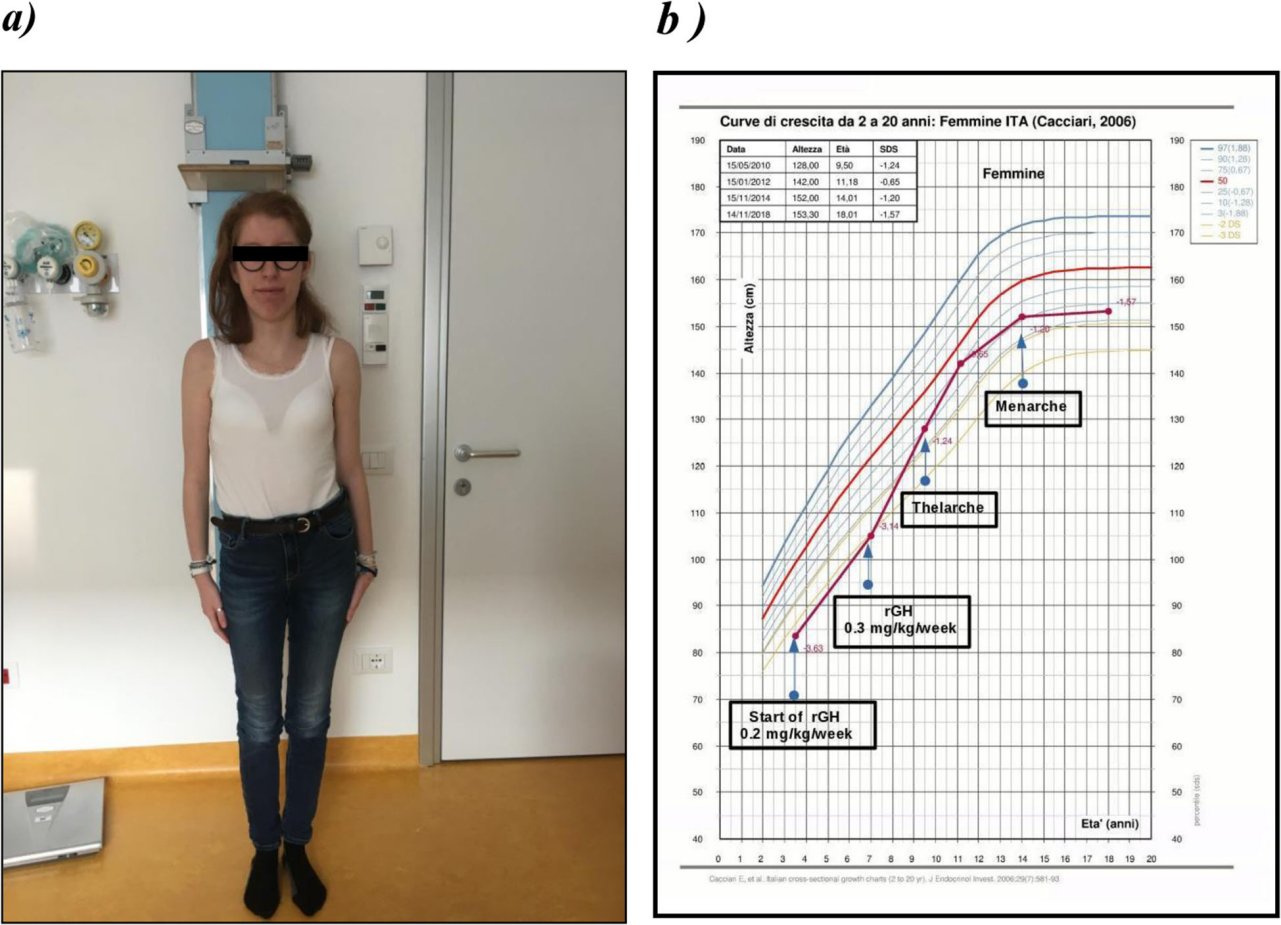
**a** Imagine of the patient with scoliosis, short stature and long face. **b** Growth curve with response to GH therapy. (Patient’s one is shown by the dot line)

When she was 18 years old the weight was 42,7 Kg (− 2 DS score) and her height was 153,2 cm (− 1 DS score): physical examination showed general facial hypotonia and nistagmus, long face with tall chin, hypotrophy of the lower extremities, widely spaced teeth, complete pubertal development (Tanner stage 5) and a moderate scoliosis for which surgery was recommended. Her follow-up shows improvement in her growth parameters (Fig. 2 a and b).

A formal cognitive evaluation was performed for the first time at 15 years old: the Wechsler Intelligence Scale for Children (WISC-III) revealing a total IQ of 77 (Verbal IQ: 81, Performance IQ: 79). Further neuropsychological evaluations highlighted the presence of a nonverbal learning disability. By age of 18 neurological evaluation remarked only a slight motor clumsiness. An electroencephalogram recorded at the same age both in wakefulness and sleep did not reveal any abnormalities. Overall, the patient’s cognitive level is sufficient to guarantee satisfactory daily activity and learning.

Laboratory test at 3rd day of menstrual cycle showed: FSH 7,8 mIU/mL, (normal 1.5–11 mIU/ml), LH 4.51 mIU/ml (normal 1–25), estradiol 104 ng/L (normal 70–530) TSH 1.28 mIU/mL (normal 0.35–3 mIU/mL), AMH 0,39 ng/dl (abnormal < 1 ng/dl) and IGF1 50 nmol/L (normal 33–62). Ultrasound examination performed by transabdominal approach showed the presence of regular uterus and ovaries with an AFC (Antral Follicle Count) of 3.

Hormonal and ultrasound examinations were compatible with a severely reduced ovarian reserve, with AMH and AFC both <5th centile for age. Considering ovarian reserve results, a counselling about available fertility preservation strategies was done. Since both the patient and her parents showed interest in this opportunity, further multidisciplinary evaluations were performed before to proceed. Risks, methods and limits of the procedure were explained, especially regarding the paucity of data about success, genetic transmission risks and the possibility to perform a Preimplantation Genetic Diagnosis (PGD) in the future. Finally, patient’s psycho-intellectual adequacy and her understanding of the proposed procedure were confirmed, and adequate time for decision was given to patient.

A cardiological examination with no contraindications detected, at time of evaluation, for future pregnancies was also performed.

The patient and her parents decided to proceed. After controlled ovarian stimulation (COS) three oocytes were obtained; two of them were in Metaphase II and were cryopreserved with vitrification. Further COS cycles are planned according to patient’s and her parents’ wills, in order to increase the number of oocytes to be preserved.

Results of these literature findings are summarized in the discussion. Our aim is to evaluate a possible correlation between genetic test results and clinic outcomes as well as to suggest possible management strategies for fertility, genetic and endocrinology specialists.

## Discussion and conclusions

In this report, we describe a patient SGA with short stature, developmental delay, mild facial dysmorphism, spontaneous puberty, DOR and Nonverbal Learning Disability. Oligonucleotide-based array comparative genomic hybridization demonstrated partial trisomy and tetrasomy Xq.

The abnormal phenotype in female with dup (X) based on selective inactivation of the aberrant dup (X) chromosome is not predictable; this phenotypic diversity may be caused by functional disomy limited to the duplicated X region, an inter-individual difference in X inactivation pattern, a tissue-dependent X activation pattern and possible incomplete inactivation of the duplicated X chromosomal segment.

Zhang et al. [22] analyzed genes that escape X-inactivation showing that the majority of them are located in the short arm and in the distal portion of the long arm of the X chromosome (a region where various genes responsible of intellectual deficit are located). Distribution of escaped genes is particularly important in polyX karyotypes because genes that are more likely to escape inactivation determine clinical symptoms related to the presence of more copies of the gene itself, while inactivated genes remain silent [22, 23] .

Although similar cases have been previously reported, clinical features of patients with Xq Duplication Syndrome are still not well defined.

Tachdjian et al. [5] reported the case of a girl with duplication Xq21.1-q25 and abnormal phenotype with growth retardation, hypotonia, and nystagmus. Carozzo et al. [15] reported a more severe case with congenital anomalies and Pelizaeus-Merzbacher disease given by PLP gene duplication. Other Authors reported cases of Xq duplication of variable extension with different phenotypic characteristics and with the presence, among others, of short stature, premature menopause and mental retardation [3, 16]. Scientific literature reported few cases with GH deficiency in Xq duplication, but all were associated to hypothyroidism or empty sella [24, 25]. No cases are reported about benefits derived from treatment with rGH in patients with Xq duplication syndrome and short stature without GH deficiency.

Our patient manifested short stature since the age of 4 years old with normal levels of IGF 1 and GH. She also had moderate scoliosis and irregular menstrual cycles with incipient ovarian failure. Scoliosis could be part of Xq duplication syndrome [8], but as in patients with TS or Prader–Willi syndrome (PWS), even in the presence of scoliosis GH therapy can be initiated or continued [26, 27]. Our patient started GH therapy and during the treatment she has always been subjected to strict medical controls of scoliosis.

Mechanisms involved in short stature are not clear and the specific genes in the duplicated region that determine height are unknown; conversely, we know that the patient did not present thyroid abnormalities or GH deficiency and that SHOX gene on Xp was normally present in two copies.

Eventually, there might be a positive correlation between the position of the X chromosome duplicated region and the growth deficit. A possible explanation is that the rearranged X chromosome with Xq duplication, could, as well as a deletion, cause alteration of chromatin structure and induce inactivation process of some genes on the X chromosome that are normally active (with possible involvement of the PAR 1 region where the SHOX gene is located and the result of its haploinsufficiency) [28].

Moreover, the number of chromosomes (or genes copies) can also affects height outcome [29]; as a matter of fact, our patient has partial trisomy and tetrasomy Xq.

In our patient, the X chromosome duplicated region contains more than 900 genes; more than 150 of them, if impaired, are responsible for known pathological pictures and many of them could determine premature ovarian failure [30, 31] and intellectual disability [32].

Among all, FMR1 and FMR2 have a double role because the expansion of their trinucleotide repetition may cause both intellectual disability and POI. Other important genes associated with ovarian failure are DACH2 (in Xq21.2), DIAPH2 (in Xq21.33) and PGRMC1 (in Xq24) [30, 33, 34]; as a consequence, it can be assumed that Xq21-Xq24 is a putative region for ovarian reserve.

Despite the presence of spontaneous menstrual cycles and normal values of FSH and LH, ovarian reserve markers such as AMH and AFC allowed detection of a compromised, even if still present, ovarian activity. At our knowledge, this is the first case of Xq duplication reporting assessment of AMH value, which is actually recognized to be the best marker of ovarian reserve in general population [35]. For these reasons, it has been proposed as a marker for early detection of ovarian failure in particular conditions such as, among others, Turner Syndrome [36–38]. Therefore, because of her risk of POI onset, a close monitoring of ovarian activity in this patient is needed [38]. Periodically evaluation recording menses characteristics and hormonal dosages are required for early detection and treatment with hormonal replacement therapy for endogenous estrogenic deficiency. As regards fertility issues, an adequate counselling is needed as well: nowadays, fertility preservation strategies such as oocyte cryopreservation and PGD are available and, even if still experimental, could be recommended in selected situations after ethical concerns have been taken into consideration and an adequate psyco-intellectual maturity [36, 39] has been assessed.

Moreover, available literature describes many cases of fertility preservation in Turner Syndrome, which could be considered a similar clinical condition [21, 39].

In conclusion, our patient presents essentially typical facies, mild intellectual disability and some features of Turner Syndrome such as reduced ovarian reserve, scoliosis and short stature. Since the latter is a very frequent condition in girls with dup Xq, it is useful to treat the patient with intensive GH therapy, even in the presence of laboratory data indicative for no GH deficiency.,

We here described for the first time a favourable response to rGH in a girl with Xq duplication. We performed an up-to date review of literature for female with Xq duplication (results are reported in Table 1). Phenotypic features reported are very variable: ovarian insufficiency and short stature are often described in these patients but AMH assessment for ovarian reserve is never reported and no patient was ever treated with rGH or preservation of fertility (Table 1).

**Table 1:** Previously published cases of Xq duplication in female (1983-present)

Authors	N.female patients	Age of patients (diagnosis and follow up)	Xq duplicated region	Clinical features	Reporting of impaired fertility status and diagnostic criteria	Fertility and short stature treatment
Present case	1	18	Xq21.31-qter	Short stature, Non verbal learning disability, mild dismorphyc features, premature ovarian insufficiency	Yes, POF defined by hornmonal (estrogens and AMH) and ultrasound criteria.	rGH theraphy and fertility preservation strategies.
Sanlaville D et al. (2009) [1]	Total 80, not known M/F ratio	Non specified	Xq21q24/Xq26qter/MECP2 duplication	Short stature, developmental delay, facial dysmorphism, gonadal dysgenesis, body asymmetry	Yes, non specified	None
Bijlma EK et al. (2012) [2]	5	3 girls, 2 adults	Xq28	Mild to moderate mental retardation, combined with variable symptoms (autistic features, recurrent infections in early childhood, constipation, and late-onset neurological features). Variables dysmorphic features	No	None
Chen CP et al. (2011) [3]	1	Adult	Xq22.1-q24	Psychomotor retardation, developmental delay, mental retardation, short stature, general muscle hypotonia, elongated digits, scanty pubic and axillary hair, hypoplastic external female genitalia, and secondary amenorrhea	Yes, POF defined by FSH and estradiolo values	None
Donnelly DE et al. (2011) [4]	1	3 year old	Xq22.3-q26	Developmental delay, slow growth, hypotonia, mild dysmorphic features (elongated face, almond shaped eyes, epicantihyc fods, broad nasal tips)	No	None
Tachdjian G et al. (2004) [5]	1	Fetus, newborn	Xq21.1-q25	Intrauterine growth retardation, SGA, hypotonia, feeding problems, poor language	No	None
Lachlan KL et al. (2004) [6]	1 female	Fetus, newborn	Xq27.1-qter	SGA, hypotonia, feeding problems, small mouth and chin, growth failure	No	None
Armostrong L et al. (2003) [7]	1	Newborn, child	Xq22.3-q26	SGA, feeding problems, variables dismorphyc features, developmental delay, mild scoliosis, short stature	No	None
Tihy F et al. (1999) [8]	1	Newborn	Xq22.1-q25	SGA, moderate psychomotor retardation, right hemiatrophy, mild scoliosis.	No	None
Correa-Cerro L et al. (1999) [9]	1	20 years old	Xq22-q23	Short stature, gonadal dysgenesis with secondary amenorrea.	Yes, POF defined by gonadotropins and estrogens values. Streak gonads in laparoscopy.	None
Monaghan KG et al. (1998) [10]	1	24 years old	Xq23-q25	Hypotonia and low weight at birth, developmental delay, compulsive behaviour, obesity, short stature, speech articulation defect, irregular menses.	No	None
Garcia-Heras J et al. (1997) [11]	1	3 year old	Xq23-q26	Growth retardation, developmental and speech delay and minor anomalies	No	None
Aughton DJ et al. (1993) [12]	1	Fetus, newborn, child	Xq13-qter	SGA, growth retardation, mild hypotonia, seizures, minor anomalies	No	None
Van Dyke et al. (1983) [13]	1	40 years old	Xq13.3–27.3	Short stature, secondary amenorrhea, and gonadal dysgenesis.	Yes, POF defined by gonadotropins values.	None
Yi Z et al. (2016) [14]	1	Child	Xq28	Developmental and speech delay, intellectual disability, feeding difficulties, seizures, recurrent infection (with fatal infection before 25 years old)	No	None
Carrozzo R et al. (1997) [15]	1	Child	Xq21.32-q24	Short stature, hypomyelination, hypotonia, ocular albinism, mild dismorphisms.	No	None
Volleth M et al. (2001) [16]	1	Adult	Xq23-q28	Dysmorphic features (simian creases, epicanthal folds, broad nasal root, retrognathia, ptosis, wide-spaced nipples), developmental delay and mental retardation. Short stature	No	None
El Chehadeh S et al. (2017) [17]	6	Variable	Xq28	Mild to moderate intellectual deficiency and no obvious dysmorphic features. Eccept one of them with a moderate to severe phenotype including very poor language, drug-resistant epilepsy, chronic constipation, stereotyped movements, behavioural disorders, recurrent infections and facial dysmorphism	No	None
Maurin ML et al. (2017) [18]	1	Fetus, newborn	Xq21.33 microduplication	Normal phenotype	No	None
Rajangam S et al. (1999) [19]	NA	NA	NA	NA	NA	NA

*NA* not available

We can conclude that females affected by Xq duplication could present very variable clinical features that must be evaluated case by case. Our patient, despite IQ at the lower limits and a Non Verbal Learning disability, carries on a challenging course of study with adequate support. For the short stature, despite absence of GH deficiency, she has been treated intensively with rGH, reaching an acceptable social height. As regards reproductive issues, she is still carefully monitored in order to promptly detect the onset of POI.

## Data Availability

Data sharing is not applicable to this article as no datasets were generated or analysed during the current study. Moreover, Authors can confirm that all clinical relevant data are included in the article and/or its additional files.

## Abbreviations

AFC: Antral Follicle Count
AMH: Anti Müllerian Hormone
COS: Controlled ovarian stimulation
DOR: Diminished ovarian reserve
PGD: Preimplantation genetic diagnosis
POF: Premature ovarian Failure (Table 1)
POI: Premature Ovarian Insufficiency
rGH: Recombinant growth hormone
SGA: Small for gestational age (Table 1)
TS: Turner Syndrome
